# Supplementary material for: Connection between f-electron correlations and magnetic excitations in UTe2
Source: NPJ Quantum Mater. 2025 Jan 4;10(1):2. doi: 10.1038/s41535-024-00720-9 (PMC11700032; doi:10.1038/s41535-024-00720-9)
Supplement: Supplementary file 1 — Supplemental Materials [file 41535_2024_720_MOESM1_ESM.pdf]

Supplementary Information: Connection between f-electron correlations and magnetic excitations in UTe_2

(Dated: November 22, 2024)

SUPPLEMENTARY NOTE 1 : EXPERIMENTAL DETAILS

The scattering experiments were performed on a coaligned mosaic of 82 single crystals of UTe_2. The crystals were mounted on a custom mount consisting of stackable plates machine from OHFC copper, pictured in Fig 1 using CYTOP polymer epoxy. All sample were aligned by using a laue diffractometer, with a sample diffraction pattern shown in Fig. 1(b). Seven of the crystals were grown by a salt flux method, with the remainder grown by more traditional chemical vapor transport (CVT). The long rod-like samples are the salt flux samples, which characteristically grow such that the long axis of the sample is along the lattice a direction. The CVT samples are rock-like, and often have an (001) facet which was used to mount, but more commonly have large (011) facets.

The CVT samples were grown using a ratio of U to Te of 5:9 by mass. The materials were sealed in argon in a quartz tube and heated 900/830 C for two weeks. The same method was used in Ref. [1] and is explained in more detail. The salt flux samples were grown in a 50:50 mixture by mass of KCl and NaCl, with starting ratios of U to Te of 1.71. These components were placed into an aluminia crucible and sealed in argon, then heated to 900 C, then slowly cooled in steps to 750 C [2]. The superconducting transition temperature T_c is $T_c \geq 1.8$ K for all samples, indicating higher sample quality than older samples with $T_c \approx$1.6 K used in previous INS measurements. While the mosaic combined many crystals from a number of batches, representative measurements of specific heat and transport are provided in Fig. 2. The sharp transition in specific heat is indicative of high sample quality, and is compared to lower temperature specific heat data from Ref. [3]

The reported mosaic misalignment in the $(hk0)$ plane of 5° is derived from sample rotation scans of the nuclear Bragg peaks. These scans are shown in Fig. 4(a-c). The full map of elastic scattering in the $(hk0)$ plane integrated in an energy window of $\hbar\omega \in (-0.1, 0.1)$ meV is shown in Fig. 3. Like the inelastic scattering, the elastic scattering is almost entirely field-independent, as shown by the difference data in Fig. 3(c). There may be small diffuse finite intensity, but it is clear from the disordered peaks that emerge at 11 T that at least one crystallite shifted from its initial position. This was likely a result of mechanical vibrations during the experiment, but accounts for a negligible amount of the spectral weight as evidenced by the nuclear Bragg peaks in Fig. 4(a,c,e). Our experiment is not well optimized for a detailed analysis of field-dependent elastic diffuse scattering, and did not find any statistically significant change.

SUPPLEMENTARY NOTE 2 : DFT+DMFT CALCULATION DETAILS

Fully charge self-consistent DFT+DMFT calculations [4–7] implemented in the WIEN2k package [8] were performed using the experimentally determined crystal structure. Continuous-time quantum Monte Carlo (CTQMC) [9, 10] was adopted as a local impurity solver. We chose a wide hybridization energy window from -10 eV to 10 eV with respect to the chemical potential. The fully rotationally invariant form was applied for the local Coulomb interaction Hamiltonian, with on-site Coulomb repulsion U=6 eV and Hund's coupling J_H=0.57 eV. The values of U and J_H explain aspects of the experimental ARPES [11] and optical conductivity [12] data. The maximum entropy method [13] was used for analytical continuation to obtain the self-energy on the real frequency axis.

SUPPLEMENTARY NOTE 3 : UPPER LIMIT ON FROZEN MOMENT

In the main text, we report an upper limit on the field-induced static moment. This is derived from the integrated Bragg intensities shown in Fig. 4. For elastic scattering from a magnetically ordered Bravais crystal, the elastic cross-section of ordered spins at a particular miller index of (hkl) may be written as [14]

$$\frac{d\sigma_{el}}{d\Omega} = N(\gamma r_0/2)^2(g\mathcal{F}(\boldsymbol{Q}_{hkl}))^2 e^{-2W} \sum_{\alpha\beta}(\delta_{\alpha\beta} - \hat{q}_\alpha \hat{q}_\beta) \sum_d \exp(i\boldsymbol{\kappa} \cdot \boldsymbol{Q}_{hkl}) \langle S_0^\alpha \rangle \langle S_d^\beta \rangle. \quad (1)$$

Where γ is the neutron gyromagnetic ratio r_0; is the classical electron radius, g is the Lande g-factor, $\mathcal{F}(\boldsymbol{Q}_{hkl})$ is the nuclear structure factor for a particular nuclear Bragg peak of index (hkl), e^{-2W} is the Debye-Waller factor which

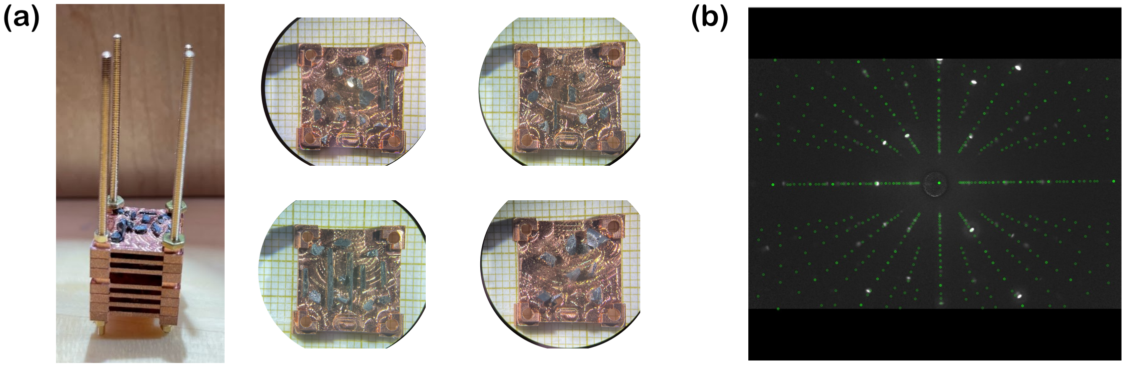

Supplementary Figure 1 : (a) Assembled mosaic of single crystal samples used in neutron scattering experiment of UTe_2 aligned such that the alignment is in the $(hk0)$ scattering plane. Four examples plates of aligned and mounted crystals are shown on the right. (b) Laue scattering pattern from one representative aligned crystal. The green dots are a calculated scattering pattern.

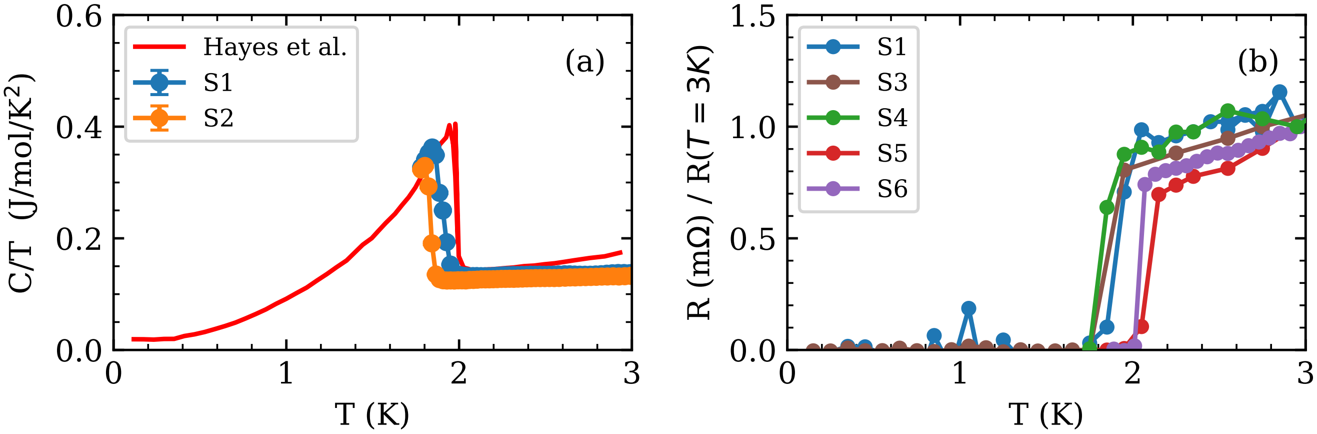

Supplementary Figure 2 : Characterization of representative samples of UTe_2 from growths used in the INS experiment. Heat capacity down to T=1.8 K is shown in (a) for two CVT batches, and transport for four CVT batches is shown in (b-e). A comparison to specific heat of a well-characterized sample from Ref. [3] is shown in (a). In the resistance curves shown in (b,c,d) were measured using an adiabatic demagnetization refrigerator insert allowing for the measurement of temperatures below T = 1.8 K, points above T=1.8 K were measured using the standard PPMS AC transport option. The highest T_c comes from the salt-flux samples, shown in (f).

we take to be one, and the summation is related to the neutron polarization factor. For a ferromagnet with many domains, we may rewrite Eq. 1 as

$$\frac{d\sigma_{el}}{d\Omega} = N(\gamma r_0/2)^2 \frac{(2\pi)^3}{V_0}(g\mathcal{F}(\boldsymbol{Q}_{hkl}))^2 e^{-2W}\{1-(\hat{Q_{hkl}}\cdot\hat{M})^2_{avg}\}\langle S^z\rangle^2. \tag{2}$$

Here, the mean-squared moment along the field-direction is $\langle S^z\rangle^2$, and the polarization summation is captured by $\{1-(\hat{Q_{hkl}}\cdot\hat{M})^2_{avg}\}=1$. We seek to put an upper bound on the field-induced magnetic moment, which we assume to be along the field direction of the c-axis. Thus, for the high field measruement the term $\{1-(\hat{Q_{hkl}}\cdot\hat{M})^2_{avg}\}=1$ as the induced moment should be along $\hat{c}$ and the scattering is in the $(hk0)$ plane. We now assume that for zero field, there is no net moment i.e. $\langle S^z\rangle^2=0$. Then, the only scattering component is the nuclear Bragg peak which has a cross section of the well known form

$$\frac{d\sigma}{d\Omega_{\boldsymbol{\tau}}} = N\frac{(2\pi)^3}{V_0}|\mathcal{F}(\boldsymbol{Q})|^2\delta(\boldsymbol{Q}-\boldsymbol{\tau})e^{-2W}. \tag{3}$$

Of course, this contribution also exists in the high field data. We may write the following convenient relation

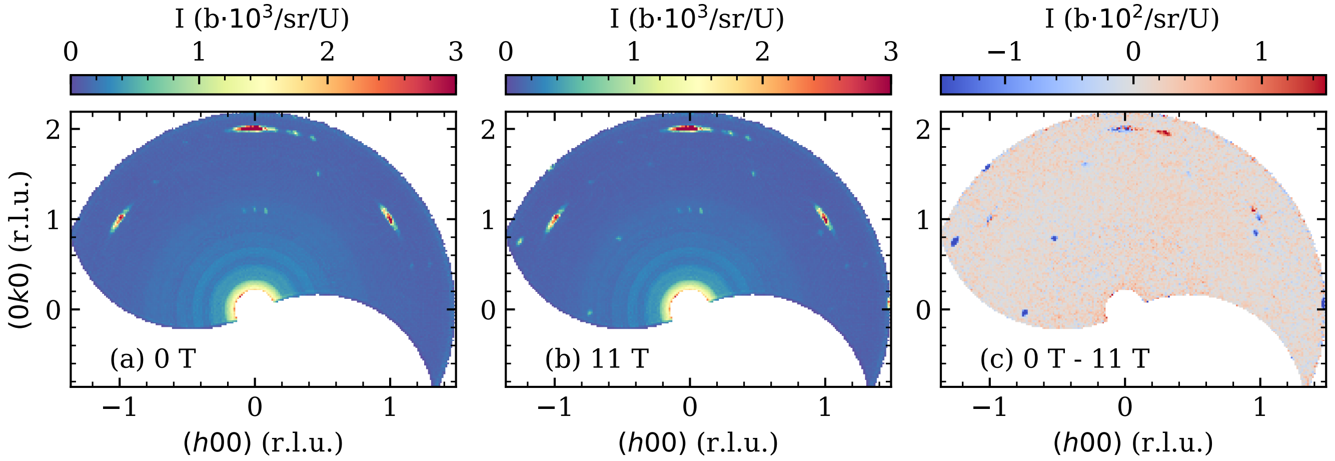

Supplementary Figure 3 : Elastic Scattering slices integrated from -0.1 meV to 0.1 meV. The zero field (a) and 11 T (b) measurements are identical, as shown by the subtracted data in (c).

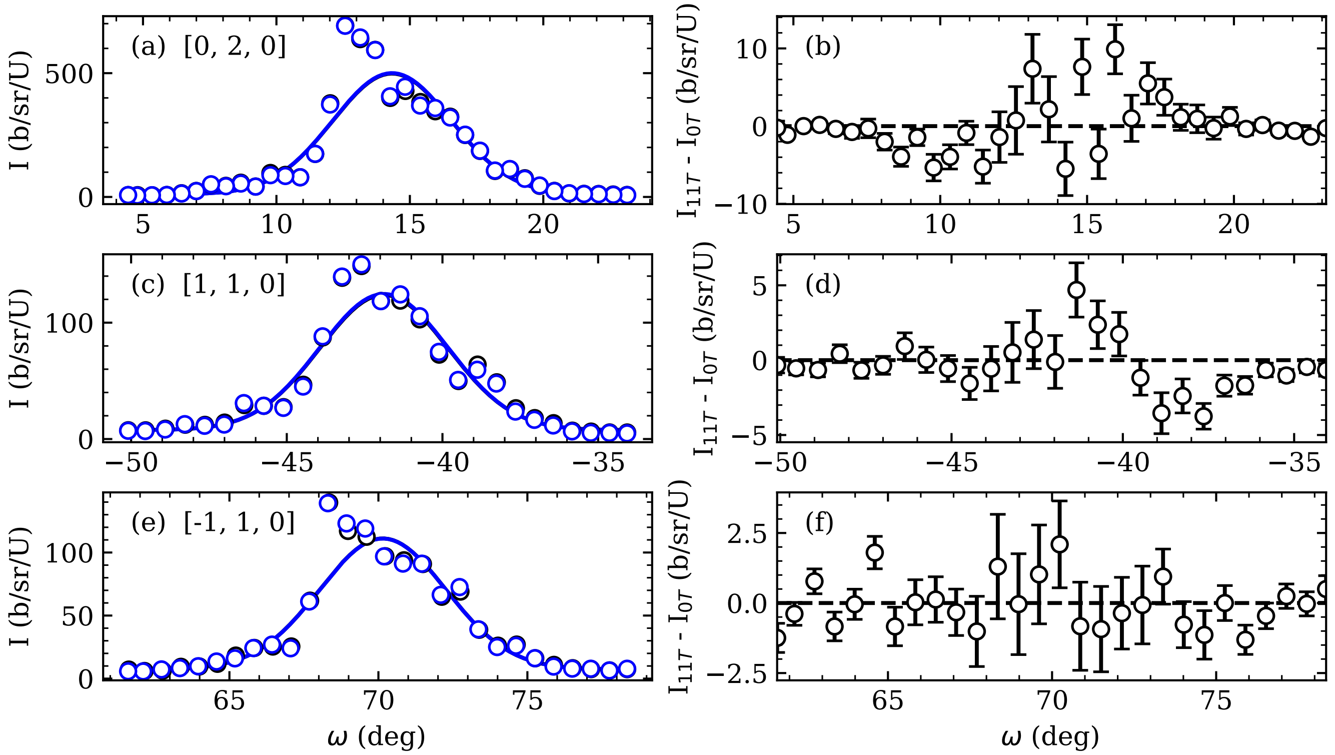

Supplementary Figure 4 : Scans over azimuthal angle of nuclear Bragg peaks. The black points in (a,c,e) are the directly measured intensities for the zero field and 11 T measurements, respectively. (b,d,e) High field 11 T scans subtracted by the zero field measurements to put an upper limit on the field-induced static moment. All error bars represent one standard deviation.

$$\mathcal{R} = \left(\frac{d\sigma_{el}^{11T}}{d\Omega} - \frac{d\sigma_{el}^{0T}}{d\Omega}\right) / \frac{d\sigma_{el}^{0T}}{d\Omega} \tag{4}$$

$$\mathcal{R} = (\gamma r_0/2)^2 g^2 \{1 - (\hat{Q_{hkl}} \cdot \hat{M})^2_{avg}\} \langle S^z \rangle^2 \tag{5}$$

Here, it is assumed that the nuclear Bragg scattering cross section is the same for both fields. We may further simplify this by noting that the term in brackets is unity, giving the final expression used of

$$\langle S^z \rangle^2 = \frac{\mathcal{R}}{(\frac{\gamma r_0}{2})^2 g^2} \tag{6}$$

In our case, the mean value of $\mathcal{R}$ from the three available Bragg peaks is about 0.003(0.05). A more careful measurement of a single crystal sample, rather than a moasic, should be able to resolve this detail more clearly, as was done in Ref. [15].

SUPPLEMENTARY NOTE 4 : BACKGROUND SUBTRACTION

The unique configuration of the CAMEA instrument allows for scattering analysis over nearly continuous energy transfers, with the standard binning step along the energy dimension being $\delta\hbar\omega = 35$ μeV. This is because of its utilization of what is known as the prismatic effect, where a position sensitive detector infers small changes in neutron energy from different Bragg reflection angles from the post-sample analyzer. Thanks to this, CAMEA grants full access to the three dimensional dynamical correlation function $S(\boldsymbol{Q}_x, \boldsymbol{Q}_y, \hbar\omega)$, where $\boldsymbol{Q}_x$ and $\boldsymbol{Q}_y$ are momentum transfer components in the scattering plane. Typically, to isolate magnetic scattering one requires a sample out background, temperature dependence, or field dependence. In our case, the scattering had no field dependence and we did not have enough time to devote to a high temperature measurement. Instead, we turn to an approach that takes advantage of CAMEA's broad coverage.

First, we make the assumption that the non-sample background only depdends on $|Q|$ and $\hbar\omega$, i.e. it is not sensitive to sample rotation and behaves like a powder. In almost all cases this is a good assumption, presuming that the sample environment has full rotational symmetry. Secondly, it must be true that all scattering from the sample obeys the symmetry of the $Immm$ space group. In the case of the $(hk0)$ plane for UTe_2, this translates to a C_{2m} symmetry. From this point, the procedure to extract a background may be summarized as the following:

1. Plot a constant energy slice integrated in an energy window large enough such that any magnetic scattering is clearly discerned by inspection. In our case, this is $\hbar\omega \in \{E_0 - 0.1, E_0 + 0.1\}$ meV, where E_0 is the mean energy of the slice.

2. Manually apply a mask to any regions with elevated intensity that obeys the C_{2m} symmetry, or are known to include magnetic scattering from previous experiments. This was made more robust by referring to previous INS results [16**?**].

3. The new masked dataset is taken to be a nonmagnetic background. A momentum averaging is performed to get a generic Q, $\hbar\omega$ dependent background,

$$I_{bkg}(Q, \omega) = \int \frac{d\Omega}{4\pi} I(\boldsymbol{Q}, \omega). \tag{7}$$

4. For every point in the measurement, the newly generated background is subtracted from the direct signal to extract the magnetic intensity.

A more common approach in triple axis experiments is to pick a region of equivalent Q to the signal of interest where one expects zero magnetic intensity, and assume it as background. This is the approach used in Refs. [15–17]. The technique used here is a natural evolution of this method, that makes use of more of the $(hk0)$ scattering plane. A schematic of this method, along with the background used in our measurement, is depicted in Fig. 5

SUPPLEMENTARY NOTE 5 : PREFERENTIAL MOMENT ORIENTATION DETAILS

Inelastic magnetic neutron scattering carries an overall prefactor to the observed intensity given by

$$\mathcal{C} \propto \sum_{\alpha\beta} (\delta_{\alpha\beta} - \hat{q}_\alpha \hat{q}_\beta). \tag{8}$$

Eq. 8 is a sum over the cartesian spin components and laboratory momentum transfer coordinates, $\alpha, \beta \in \{x, y, z\}$. As neutrons are only sensitive to the perpendicular component of the magnetic moment to the momentum transfer, one may rewrite Eq. 8 as

$$\mathcal{C} \propto (1 - \frac{(\boldsymbol{Q} \cdot \hat{M})^2}{|Q|^2}). \tag{9}$$

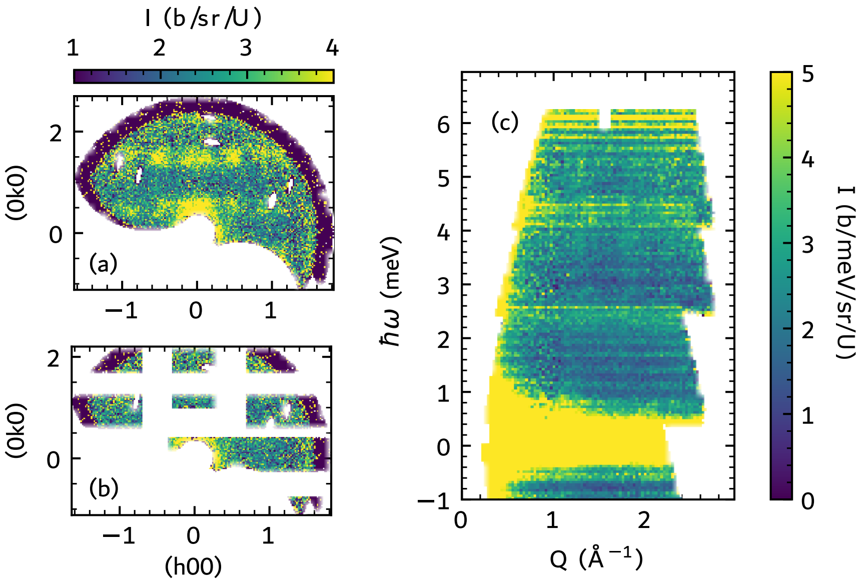

Supplementary Figure 5 : Demonstration of background subtraction routine used on CAMEA. (a) Constant energy slices integrated in energy from $\hbar\omega$=1.8 meV to $\hbar\omega$=2.2 meV. (b) The same slice, but with regions in the scattering plane identified as contributing to the scattering signal masked. (c) The powder average of the masked data, which is subtracted from the reported measurement.

Here $\boldsymbol{Q}$ is the observed momentum transfer and $\hat{M}$ is the Cartesian spin orientation. In an isotropic system such as an uncorrelated paramagnet this factor averages to $\frac{2}{3}$. In the case of UTe_2, it is well known that the a-axis in UTe_2 is the easy-axis from magnetization studies [18], so we assume that the spins prefer to align along the lattice $\hat{a}$ vector despite the system lacking long-ranged magnetic order. This is also suggested by previous neutron studies [19] which reach a similar conclusion. As no magnetic phase transition is observed in UTe_2, we may only refine an average spin orientation rather than a distinct magnetic ordered moment. This average is denoted as θ_a, which is the deviation of the moment from the $\hat{a}$-lattice vector. The results are summarized in Fig. 6, showing the calculated scattering for various moment directions using Eq.3 in the main text. No signficant difference was found when allowing for a finite moment component along the $\hat{c}$-lattice vector, apart from an upper limit on the spin angle from the $\hat{a}$-axis towards the $\hat{c}$-axis of 35°.

SUPPLEMENTARY NOTE 6 : LOW ENERGY EXCITATION SPECTRA

The main text is focused on the excitation spectra relevant to the band hybridization, where scattering is clearly seen at energy transfers within $\hbar\omega \in \{1, 6\}$ meV. Previous reports have found evidence of a spin-resonant excitation, typically assosciated with spin-singlet superconductors, at energies of 1 meV at the Y_1 (0,0.6,0) and Y_2 (0,1.4,0) points [15, 16]. We are unable to reproduce this excitation in our inelastic scattering. To demonstrate this more clearly, the scattering intensity versus energy transfer with and without background subtraction is shown in Fig. 7. As the scattering in Ref. [16] was reported in absolute units, no scaling factors are required to directly compare the measurements provided that the same integration window is used. Both measurements are compatible within the ranges of the error both above and below the renergies of the resonant excitation.

[1] C. E. Frank, S. K. Lewin, G. S. Salas, P. Czajka, I. Hayes, H. Yoon, T. Metz, J. Paglione, J. Singleton, and N. P. Butch, Orphan High Field Superconductivity in Non-Superconducting Uranium Ditelluride (2023), arXiv:2304.12392 [cond-mat].

[2] H. Sakai, P. Opletal, Y. Tokiwa, E. Yamamoto, Y. Tokunaga, S. Kambe, and Y. Haga, Single crystal growth of superconducting ${\mathrm{UTe}}_{2}$ by molten salt flux method, Physical Review Materials **6**, 073401 (2022), publisher: American Physical Society.

[3] I. M. Hayes, T. E. Metz, C. E. Frank, S. R. Saha, N. P. Butch, V. Mishra, P. J. Hirschfeld, and J. Paglione, Robust nodal behavior in the thermal conductivity of superconducting UTe_2 10.48550/arXiv.2402.19353 (2024), arXiv:2402.19353.

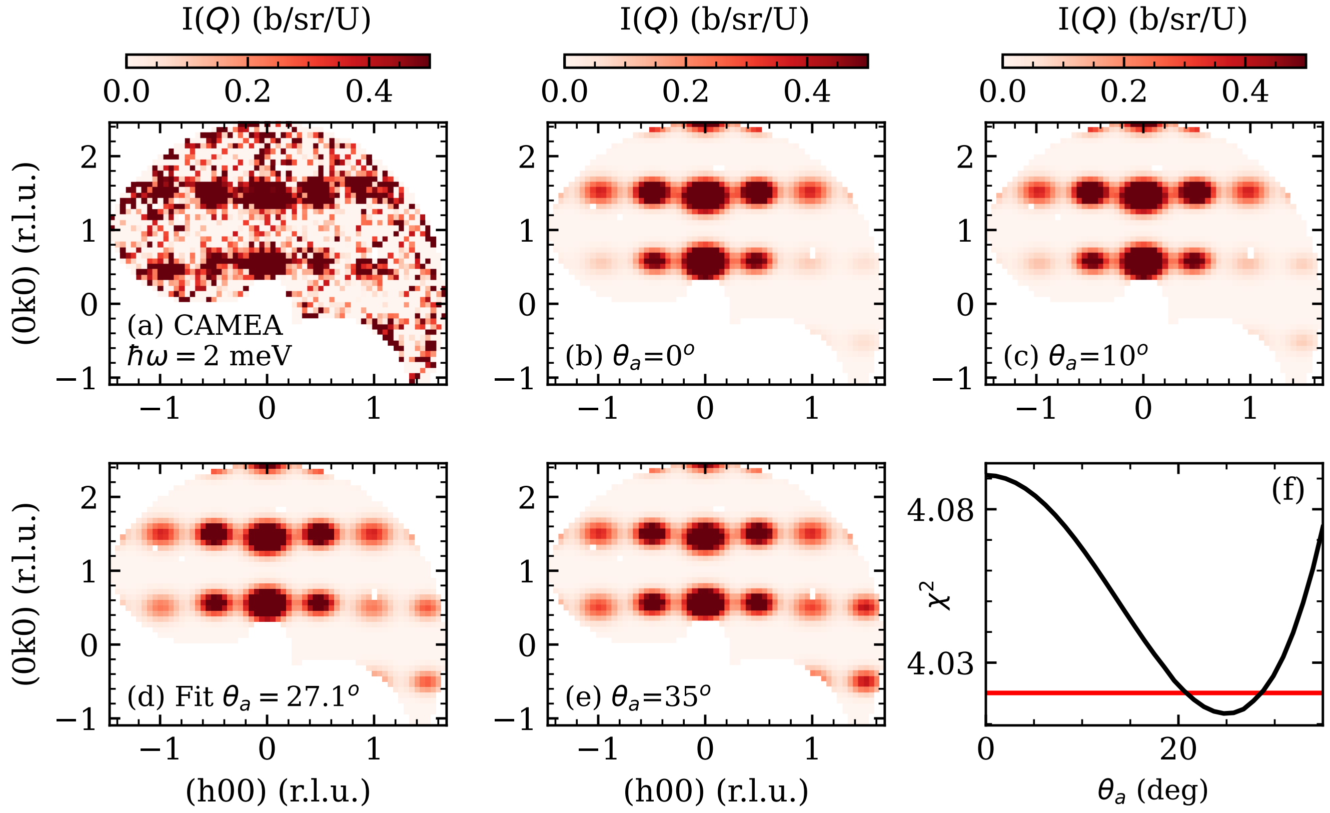

Supplementary Figure 6 : (a) Unsymmetrized integrated scattering in the $(hk0)$ plane integrated in energy from $\hbar\omega \in \{1.7, 2.3\}$ meV. (b-e) Calculated scattering using the model described by Eq. 3 in the main text, for various values of θ_a, where θ_a is the angular deviation of the magnetic moment from the $\hat{a}$-axis, and the moment is constrained to be in the ab-plane. The best fit value of θ_a=16.7 degrees is shown in (d). (f) Variation of χ^2 goodness of fit parameter versus θ_a, where all other fit parameters are freely refined for every θ_a. The red line represents the maximum value of χ^2 from which the error bar is defined.

[4] A. Georges, G. Kotliar, W. Krauth, and M. J. Rozenberg, Dynamical mean-field theory of strongly correlated fermion systems and the limit of infinite dimensions, Reviews of Modern Physics **68**, 13 (1996).

[5] G. Kotliar, S. Y. Savrasov, K. Haule, V. S. Oudovenko, O. Parcollet, and C. Marianetti, Electronic structure calculations with dynamical mean-field theory, Reviews of Modern Physics **78**, 865 (2006).

[6] K. Held, Electronic structure calculations using dynamical mean field theory, Advances in physics **56**, 829 (2007).

[7] K. Haule, C.-H. Yee, and K. Kim, Dynamical mean-field theory within the full-potential methods: Electronic structure of $CeIrIn_5$, $CeCoIn_5$, and $CeRhIn_5$, Physical Review B—Condensed Matter and Materials Physics **81**, 195107 (2010).

[8] P. Blaha, K. Schwarz, F. Tran, R. Laskowski, G. K. Madsen, and L. D. Marks, Wien2k: An apw+ lo program for calculating the properties of solids, The Journal of chemical physics **152** (2020).

[9] P. Werner, A. Comanac, L. De'Medici, M. Troyer, and A. J. Millis, Continuous-time solver for quantum impurity models, Physical Review Letters **97**, 076405 (2006).

[10] K. Haule, Quantum monte carlo impurity solver for cluster dynamical mean-field theory and electronic structure calculations with adjustable cluster base, Physical Review B—Condensed Matter and Materials Physics **75**, 155113 (2007).

[11] L. Miao, S. Liu, Y. Xu, E. C. Kotta, C.-J. Kang, S. Ran, J. Paglione, G. Kotliar, N. P. Butch, J. D. Denlinger, *et al.*, Low energy band structure and symmetries of UTe_2 from angle-resolved photoemission spectroscopy, Physical review letters **124**, 076401 (2020).

[12] S. M. Mekonen, C.-J. Kang, D. Chaudhuri, D. Barbalas, S. Ran, G. Kotliar, N. P. Butch, and N. P. Armitage, Optical investigation of the heavy-fermion normal state in superconducting UTe_2, Physical Review B **106**, 085125 (2022), publisher: American Physical Society.

[13] M. Jarrell and J. E. Gubernatis, Bayesian inference and the analytic continuation of imaginary-time quantum monte carlo data, Physics Reports **269**, 133 (1996).

[14] G. L. Squires, *Introduction to the theory of thermal neutron scattering*, Vol. 9781107644069 (Cambridge University Press, 2012) publication Title: Introduction to the Theory of Thermal Neutron Scattering.

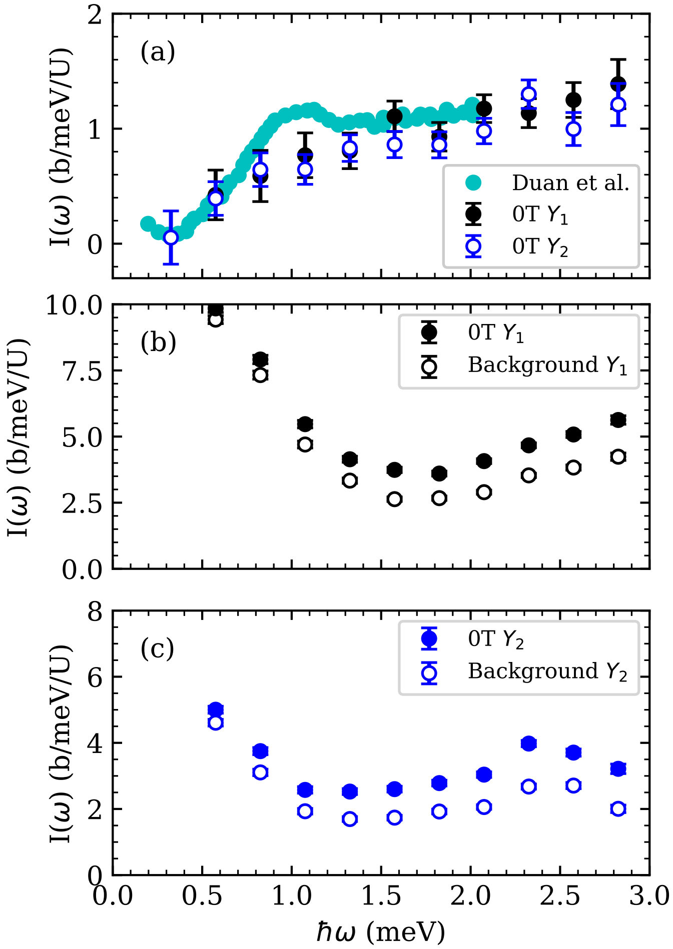

Supplementary Figure 7 : Constant momentum cuts integrated in circular windows in $\boldsymbol{Q}$ of diameter 0.2 $Å^{-1}$. The cuts are at the Y_1=(0,0.6,0) and Y_2=(0,1.4,0) points, where excitations have been reported in Refs. [15, 16]. (a) Cuts at both positions as a function of energy after background subtraction. No significant peak is observed in our data. The normalized data reported in Ref. [16] are plotted by the cyan points, where no scaling factors have been applied as both measurements are in absolute units. The same cuts of the directly measured intensity at the Y_1 (a) and Y_2 (b) points is shown along with the extracted background for each of these positions.

[15] S. Raymond, W. Knafo, G. Knebel, K. Kaneko, J.-P. Brison, J. Flouquet, D. Aoki, and G. Lapertot, Feedback of Superconductivity on the Magnetic Excitation Spectrum of UTe2, Journal of the Physical Society of Japan **90**, 113706 (2021), publisher: The Physical Society of Japan.

[16] C. Duan, R. E. Baumbach, A. Podlesnyak, Y. Deng, C. Moir, A. J. Breindel, M. B. Maple, E. M. Nica, Q. Si, and P. Dai, enResonance from antiferromagnetic spin fluctuations for superconductivity in UTe2, Nature **600**, 636 (2021), number: 7890 Publisher: Nature Publishing Group.

[17] C. Duan, K. Sasmal, M. B. Maple, A. Podlesnyak, J.-X. Zhu, Q. Si, and P. Dai, Incommensurate Spin Fluctuations in the Spin-Triplet Superconductor Candidate UTe_2, Physical Review Letters **125**, 237003 (2020), publisher: American Physical Society.

[18] S. Ran, C. Eckberg, Q.-P. Ding, Y. Furukawa, T. Metz, S. R. Saha, I.-L. Liu, M. Zic, H. Kim, J. Paglione, and N. P. Butch, Nearly ferromagnetic spin-triplet superconductivity, Science **365**, 684 (2019), publisher: American Association for the Advancement of Science.

[19] W. Knafo, G. Knebel, P. Steffens, K. Kaneko, A. Rosuel, J.-P. Brison, J. Flouquet, D. Aoki, G. Lapertot, and S. Raymond, Low-dimensional antiferromagnetic fluctuations in the heavy-fermion paramagnetic ladder compound UTe_2, Physical Review B **104**, L100409 (2021), publisher: American Physical Society.
